# What makes the sinoatrial node tick? A question not for the faint of heart

**DOI:** 10.1098/rstb.2022.0180

**Published:** 2023-06-19

**Authors:** Lorenzo Donald, Edward G. Lakatta

**Affiliations:** National Institutes of Health, National Institute on Aging, Intramural Research Program, Baltimore, MD 21224, USA

**Keywords:** calcium, pacemaker cell, telocyte, sinoatrial node, rhythm, local calcium releases

## Abstract

Even before the sinoatrial node (SAN) was discovered, cardiovascular science was engaged in an active investigation of when and why the heart would beat. After the electrochemical theory of bioelectric membrane potentials was formulated and the first action potentials were measured in contracting muscle cells, the field became divided: some investigators studied electrophysiology and ion channels, others studied muscle contraction. It later became known that changes in intracellular Ca^2+^ cause contraction. The pacemaking field was reunited by the coupled-clock theory of pacemaker cell function, which integrated intracellular Ca^2+^ cycling and transmembrane voltage into one rhythmogenic system. In this review, we will discuss recent discoveries that contextualize the coupled-clock system, first described in isolated SAN cells, into the complex world of SAN tissue: heterogeneous local Ca^2+^ releases, generated within SAN pacemaker cells and regulated by the other cell types within the SAN cytoarchitecture, variably co-localize and synchronize to give rise to relatively rhythmic impulses that emanate from the SAN to excite the heart. We will ultimately conceptualize the SAN as a brain-like structure, composed of intercommunicating meshworks of multiple types of pacemaker cells and interstitial cells, intertwined networks of nerves and glial cells and more.

This article is part of the theme issue ‘The heartbeat: its molecular basis and physiological mechanisms’.

## 1. Introduction

The enigma of when and why our next heartbeat will occur is a question not for the ‘faint of heart’ that has *gradually* been tackled both prior to and since the discovery of the sinoatrial node (SAN) 116 years ago [1].

Since the mid-nineteenth century, the debate on the origin of the heartbeat has been centred around two contrasting hypotheses: the neurogenic theory, which postulates nerves to be responsible for contractile timing; and the myogenic theory, which hypothesizes that a portion of heart muscle itself as capable of beating spontaneously and rhythmically [2]. Both these hypotheses are based on studies on bioelectricity, which, by the late-1800s, had established a causal link between electrical activity and muscle contraction (EC coupling) thanks to the invention of the galvanometer. By the turn of the century the ‘membrane theory' of bioelectric potentials had been developed to explain the mechanism by which cells manipulate their transmembrane voltage (*V*_m_), which was quantitatively proven in 1912 through the application of the Nernst equation (for review see [3]). The invention of microelectrodes in the 1950s paved the way for the Nobel Prize-winning description of action potential (AP) generation as based on selective ion flow across membranes via voltage-gated channels of giant squid axons [4]. Silvio Weidmann joined the effort to solve the enigma of how changes in membrane potential were linked to contraction. Weidmann applied the microelectrode technique to canine cardiac muscle, which enabled him to provide the first description of the inotropic effect of current injection into muscle cells, as well as the unique broader shape of cardiac muscle APs [5]. From the mid-1950s, the studies of pacemaker electrophysiology and cardiac contractility began to advance along parallel, but nonetheless separate, courses, each using distinct tools and techniques to answer its respective questions (for review see [6]).

In the late 1960s, Silvio Weidmann again entered the picture, helping to unify the studies of membrane potentials, ion currents and Ca^2+^. In their seminal work Wood, Heppner & Weidmann formulated possible mechanisms for ‘*memory*' formation within cardiac myocytes informed by experiments employing the voltage clamp, tension sensors and manipulation of the rate and pattern of electrical stimulation, as well as controlled changes of free extracellular Ca^2+^ in small bundles of bovine ventricular muscle (preparation developed by Kavaler [7]) [8]. One of the possibilities they envisioned was that Ca^2+^ storage and release from the sarcoplasmic reticulum (SR) mediated the contraction amplitude within a given beat, based on the patterns of beats that preceded it [6,9,10]. Eventually, the process of Ca^2+^-induced Ca^2+^ release (CICR) from the SR was uncovered, explaining how the inward L-type Ca^2+^ current of cardiac APs triggered sufficient Ca^2+^ release from the SR to activate myofilaments [11]. The discovery of ryanodine receptors (RyRs), Ca^2+^ channels on the SR and their antagonist ryanodine, catalysed the study of intracellular Ca^2+^ flux in cardiomyocyte contraction [12]. Ultimately, it was discovered that the SR of ventricular myocytes can generate spontaneous, roughly rhythmic Ca^2+^ oscillations [13–16]. This led to the idea that spontaneous local diastolic Ca^2+^ releases might be implicated in the normal automaticity of the SAN [17].

Here, we will examine the evolution of knowledge over the past few decades regarding how local Ca^2+^ releases (LCRs) function within the coupled-clock system of SAN pacemaker cells studied in isolation. Our main goal is not to provide a comprehensive listing of specific molecules and biophysical mechanisms that are involved in single, isolated pacemaker cell activity. Rather, it is to review and discuss evidence that underpins a major paradigm shift in SAN function.

We will first describe how LCRs that are heterogeneous within and among loci of isolated pacemaker cells emerge during the decay of AP-induced calcium transients, even prior the development of the subsequent maximum diastolic membrane potential [18]. Thereafter, the self-ordering (partial synchronization) of LCRs, concurrent with activation of numerous plasma membrane Ca^2+^ channels (e.g. Ca_v_1.3 and Ca3.1), creates a common electrochemical (Ca^2+^ + *V*_m_) signal that produces the spontaneous diastolic depolarization, which increases, in a feed-forward, nonlinear manner, to culminate in the rapid upstroke of an AP followed by CICR in SAN pacemaker cells [19].

Next, we will describe how novel 3D confocal immunohistochemical studies of SAN cytoarchitecture have uncovered complex, brain-like arrangements of multiple pacemaker cell types as well as neurons, glial cells and more [20,21]. We will then discuss how this complex cytoarchitecture contextualizes the recent discovery that LCRs are heterogeneous in phase, frequency and amplitude, not only within individual cells, but also among pacemaker cells embedded in SAN tissue.

Ultimately we will address how synchronized SAN impulses can now be understood to emerge from the self-ordering of spontaneous, heterogeneous, subcellular, subthreshold Ca^2+^ signals within and among pacemaker cells, paralleling multiscale complex processes of impulse generation in cell clusters within neuronal networks.

## 2. A coupled-clock system drives action potential firing in single sinoatrial node cells

**Figure 1.**
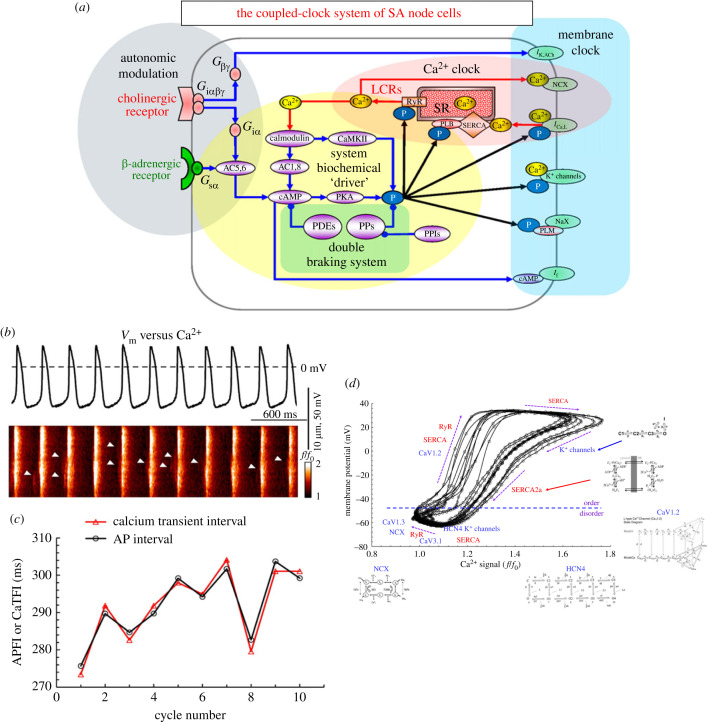
(*a*) A diagram describing the coupled-clock system described in isolated SAN cells, including autonomic modulation and the intracellular signal cascades it impacts. (*b*) The *V*_m_ (mV, measured by patch clamp) and Ca^2+^ (*f*/*f*_0_, imaged by line-scan microscopy) domain parameters recorded simultaneously in a 10 beat time series. (*c*) The AP and AP-induced Ca^2+^ transient firing intervals (APFI) within the time series in (*b*). (*d*) The phase diagram of inter-AP intervals measured in a single rabbit SAN cell, with membrane potential plotted as a function of Ca^2+^ over the course of each beat within the time series in (*b*). The times at which membrane channels are activated throughout the cycle are indicated in blue, and the sarcoplasmic reticulum (SR) channels are indicated in red. The directions of transmembrane voltage changes and AP-induced Ca^2+^ transient development are shown by the violet arrows. Activation diagrams of the most relevant coupled-clock proteins are also shown. The dashed line in the figure marks the approximate boundary between what are considered to be the ordered and disordered phases of the cycle. (*a*) Adapted from [26]; (*b*,*c,d*) adapted from [27].

The use of confocal microscopy to image spontaneous Ca^2+^ activity with fluorescent indicators in pacemaker cells enabled the discovery that isolated SAN cells exhibit rhythmic LCRs [22,23]. Later, it was discovered that LCRs can occur independently of membrane potential changes, and that in intact SAN cells LCRs are involved in the initiation of APs [24] by activating the sodium–calcium exchanger (NCX), leading to cell membrane depolarization, which consequently activates the ensemble of ion channels on the membrane of the cell (electronic supplementary material, videos S1*a*,*b*) [22,25]. Thus, functions of this ‘Ca^2+^ clock' and those of a ‘membrane clock,' composed of surface membrane electrogenic molecules, are intertwined to form a ‘coupled-clock’ system (figure 1*a*) that generates spontaneous AP cycles in SAN cells, i.e. SAN automaticity (for detailed information on coupled-clock system molecules, see [25,28–32]). In brief, in figure 1*a* the ‘membrane clock' refers to molecules located on the cell surface membrane, and the ‘Ca^2+^ clock’ refers to molecules associated with the SR. In addition to molecules that regulate ion-flux, a number of biochemical drivers are crucial to the pacemaker cell coupled-clock system. Low-voltage activated Ca^2+^ channels (Ca_v_1.3), T-type Ca^2+^ channels (Ca_v_3.1) and L-type Ca^2+^ channels (Ca_v_1.2), as well as *I*_NCX_ are members of both clocks. Recent experimental evidence and novel numerical modelling describe how *nonlinear, feed-forward interactions* among these molecular functions, and LCRs generated via RyRs, underlie spontaneous diastolic depolarization, as opposed to specific single mechanisms being ‘responsible for’ spontaneous rhythmic APs [19]. Note also that autonomic receptor activation modulates the performance of the coupled-clock system via impacting on molecules of both clocks.

The ability of the SR ‘Ca^2+^ clock' to generate spontaneous LCRs within SAN pacemaker cells is conferred by their constitutively active cyclic adenosine monophosphate/protein kinase A (cAMP/PKA) regulatory elements, which enable it to drive rhythmic cellular functions even in the absence of β-adrenergic receptor activation by nervous input. In particular, basal Ca^2+^/calmodulin-dependent adenylyl cyclase [33] leads to increased levels of cyclic AMP, which upregulates cAMP-gated ion channels, as well as activation of protein kinase A and Ca^2+^/calmodulin-dependent kinase II, both of which ultimately modulate intracellular Ca^2+^ dynamics and transmembrane potential in SAN cells (figure 1*a*) [30]. The increased Ca^2+^-dependent phosphorylation of both Ca^2+^ and membrane clock proteins that follows from the workings of this ‘biochemical engine' is *crucial* for the spontaneous transitions in subcellular Ca^2+^ and transmembrane voltage [34] that drive the ‘ignition phase onset' of the pacemaker cell AP cycle during spontaneous diastolic depolarization [19]. This is that time during the AP cycle at which evidence for interactions between Ca^2+^ and electrogenic cell surface molecules becomes first notable in membrane potential recordings. The positive feedback that occurs next between intracellular Ca^2+^ and surface membrane electrogenic molecules creates the nonlinearity of the diastolic membrane depolarization, reaching criticality, i.e. the rapid upstroke of the AP.

Spontaneous, rhythmic, phosphorylation-dependent, oscillatory Ca^2+^ releases from the SR (Ca^2+^ clock) via its Ca^2+^ release channels, i.e. RyRs, result in the appearance of LCRs during the diastolic phase of the AP cycle of pacemaker cells (figure 1*b*). LCRs propagate as Ca^2+^ wavelets for distances of up to approximately 10 µm from their subcellular loci of origin and activate the Ca^2+^-dependent electrogenic NCX channels on the surface of the cell (cuing the membrane clock), which, in turn, generate an inward electric current leading to local depolarization [22]. As LCRs begin to spontaneously occur at additional loci throughout the cell, they summate (self-organize) into a stronger Ca^2+^ signal, which leads to an abrupt acceleration in membrane depolarization referred to as the ‘ignition phase onset' [19]. The amplitude, size and frequency of LCRs increases as membrane depolarization cues the opening of low-voltage activated Ca^2+^ channels (Ca_v_1.3 and Ca_v_3.1). This positive feedback during the generation of this electrochemical (Ca^2+^–*V*_m_) signal imparts nonlinearity to the diastolic membrane depolarization, causing it to accelerate towards criticality, as the level of membrane potential depolarization becomes sufficient for the activation of voltage-gated L-type Ca^2+^ channels. This generates the rapid upstroke of the AP [19,35]. Ca^2+^ influx through L-type Ca^2+^ channels then initiates synchronized cell-wide CICR from the SR via RyRs—an AP-induced cytosolic Ca^2+^ transient. Activation of potassium channels and the decay of the AP-induced calcium transient occur during repolarization of the cell membrane [27]. It is important to note that the SR does not only pump Ca^2+^ during the decay of the AP-induced calcium transient, but does so *continuously* throughout the cycle, and that the speed at which it pumps Ca^2+^ is Ca^2+^-dependent. The increase in cytosolic Ca^2+^ during the AP-induced calcium transient upregulates the kinetics of SERCA2a Ca^2+^ pumping into the SR, causing the cytosolic Ca^2+^ concentration to decay and the SR to reload. Concurrent voltage- and current-clamp experiments in human embryonic stem cell-derived cardiomyocytes demonstrated that, although the expression of proteins for each clock is heterogeneous among SAN cells, pharmacologically blocking either clock leads to a cessation of AP generation, implying that both are essential in these cultured cells [36]. More recently, single nucleus RNA sequencing quantified a significantly higher difference in the expression of membrane clock ion channels than in the expression of Ca^2+^ clock proteins among sinoatrial cells and cells from the surrounding atrium [37,38].

But how do clock proteins, with variable activation states and synchronicity, work together to *reliably* generate AP cycles? The answer lies in their activation cues: activation of some clock molecules is Ca^2+^-dependent, while activation of others is *V*_m_-dependent, and activation of some clock molecules depends on both Ca^2+^ and *V*_m_ (figure 1*a*). Ca^2+^ and *V*_m_ transitions occurring throughout AP cycles in SAN cells not only *regulate* the activation states of molecules operating within the Ca^2+^ and *V*_m_ domain, but are also *regulated by* the activation states of the clock molecules they regulate. In other terms, these cues are both causes and effects (recursion) of clock molecular activation [27]. An excellent example of this relationship is the NCX, responsible for trafficking ions across the cell membrane to depolarize it during diastole. The NCX protein structure contains allosteric Ca^2+^ binding domains that regulate its activity so it is stimulated by LCRs, which means it both regulates and is regulated by intracellular Ca^2+^ concentration [22,39]. The phase-plane diagram in figure 1*d* depicts how simultaneously measured changes in cytosolic Ca^2+^ and transmembrane voltage during AP cycles cue the activation states of multiple pre-eminent Ca^2+^- and *V*_m_-dependent molecules, including NCX [27]. This recursive relationship occurring throughout the pacemaker cell AP cycle is what confers robustness and reliability to the coupled-clock dynamic on a beat-to-beat basis [30].

Recent studies on the kinetic parameters of beating rate in both isolated SAN cells and *in vivo* animals have provided evidence that the basic nature of SAN automaticity does not differ among species (electronic supplementary material, videos S1*a*,*b*) [26]. The AP cycle lengths, as well as the rates of rise and decay of APs and AP-induced calcium transients, measured by confocal and line-scan microscopy during the AP cycles of cells isolated from mouse, guinea pig, rabbit and human SANs demonstrated long-range correlations with species body size with nearly the same (body mass^0.25^) scaling exponent as observed previously [26]. The same study found that QT, RR and PR intervals obtained by electrocardiogram (EKG) recordings from these species scaled with the kinetic parameters mentioned above, indicating that the specific parameters of heart rhythm generation scale with body mass and target heart rate across species both in isolated cells and in live animals [26]. This consistency across species lends credibility to the idea that experiments on animal models such as mice yield results that can be interpreted as applicable across species [26].

The activation states of individual clock proteins, as well as the degree of coupling of the activation levels of clock proteins, can be modified over the time course of multiple beats by post-translational modifications, which can be altered by both the intra- and extracellular context to suit the body's needs for faster or slower AP firing rate. For example, RyRs, phospholamban and L-type Ca^2+^ channels are among the clock proteins the activity of which is enhanced by PKA-dependent phosphorylation, which can be impacted upon by neurotransmitter activation of autonomic receptors or changes in cytosolic Ca^2+^ concentration (figure 1*a*). Constitutive phosphodiesterase and phosphoprotein phosphatase activity counterbalances the constitutively active adenylyl cyclase in SAN cells, to maintain the equilibrium of clock protein phosphorylation associated with each quasi-steady-state mean AP firing rate required for different levels of cardiac performance [26,40]. Ventricular myocytes, despite their lack of spontaneous rhythmic activity, express many of the same molecules that function within the coupled-clock system of SAN pacemaker cells. In ventricular myocytes, however, these proteins exhibit lower levels of phosphorylation relative to SAN cells [34]. It is, therefore, noteworthy that spontaneous LCR-triggered APs emerge in ventricular cardiac myocytes when phosphorylation of these molecules is increased via inhibition of phosphodiesterases and phosphoprotein phosphatases [41].

AP firing behaviour ranges from rhythmic activity, defined by the most rapid rates and lower AP variability, to ‘dormant' cells characterized by smaller highly stochastic LCRs. Rhythmic activity occurs when phosphorylation states are high and LCR intervals are the lowest (e.g. during β-adrenergic receptor stimulation). Dysrhythmic AP firing at slower rates is associated with highly variable inter-AP intervals, and is seen when phosphorylation of clock proteins is low (e.g. during cholinergic receptor activation). Dormancy is associated with extremely low phosphorylation states [42,43]. Dysrhythmic or dormant cells can be recruited by β-adrenergic stimulation, which increases clock protein phosphorylation levels, increasing the degree of synchronization of LCRs, resulting in rhythmic AP firing [44]. Thus, the range of phosphorylation states of clock proteins, and therefore the degree of synchronization of molecular activation states across the coupled-clock system, underlies the full spectrum of SAN cell AP firing rates and rhythms that can be observed in isolated SAN cells [27,30].

Constant changes in the intra- and extracellular dynamics that influence clock protein activation states and clock molecule coupling result in the system never reaching steady-state equilibrium, which is the cause of the well-known variability in the AP firing intervals of isolated SAN cells (figure 1*c*) [27], as well as SAN tissues *ex vivo*, and in the *in vivo* heart rate of animals and humans [45–48]. The long- and short-term variability in the intervals between cardiac APs, in the range of milliseconds around the mean beating rate, is indicative of the Ca^2+^- and phosphorylation-dependent memory encoded within the clock proteins and manifest as variable coupling between the clocks [27]. This is consistent with the understanding that higher beating rates, known to correspond to higher clock-coupling, are also correlated with lower heart rate variability, and lower beating rates with higher variability [47]. Thus, the body influences the rate and rhythm of SAN pacemaking via autonomic receptor activation, which impacts on the activation states and coupling of the *same proteins* that are involved in the coupled-clock system in the absence of autonomic neurotransmitter input (figure 1*a*).

## 3. The role of *I*_f_ in the coupled-clock system of sinoatrial node pacemaker cells

Prior to the appreciation of how a coupled-oscillator system underlies normal SAN cell automaticity, it had been ‘in vogue' to attribute normal automaticity to a single, or most important, surface membrane ion channel current. The hyperpolarization-activated inward current (*I*_f_), conducted via hyperpolarization-activated cyclic nucleotide-gated channels (HCN), was identified as a prime candidate to be the pacemaker current of SAN cells. Because the activation of HCN channels is positively regulated by cAMP, the increase in heart rate in response to β-adrenergic receptor stimulation had been attributed to their increased activation [49,50]. However, genetic ablation of the HCN4 channel [51], its modification to make it insensitive to cAMP [52], direct pharmacological inhibition of *I*_f_ [51], as well as its reduction in parametric sensitivity analyses in numerical models [53], all result in sinus bradycardia or arrest and chronotropic incompetence [54], without having much of an effect on the β-adrenergic receptor stimulation-induced increase in the AP firing rate of the coupled-clock system. These modern perspectives have led to the viewpoint that, rather than being essential for *increasing* the AP firing rate of pacemaker cells, *I*_f_ is required to ensure coupled-clock signalling during the AP ignition phase [19], because it prevents membrane potential from hyperpolarizing to a degree that compromises clock signalling during the diastolic ignition phase. In other terms, the critical contribution of *I*_f_ to SAN cell coupled-clock system function is that its inward rectification prevents membrane potential repolarization to the theoretical electrochemical equilibrium of potassium channels.

## 4. From single pacemaker cells to sinoatrial node tissue

### (a) Early studies

It was recognized that sinoatrial nodal pacemaker cells were defined by markedly lower myofilament density than their surrounding atrial myocytes, counterbalanced by higher expression of ion channels [55]. The use of transfer electron microscopy enabled the identification of a subpopulation of smaller SAN cells exhibiting distinct intracellular organelle organization characterized by a lower density of myofilaments, leading to the theory that these were ‘leading pacemaker cells', whose cellular organization deprioritized contractile for rhythmogenic architecture [55,56].

Electrical mapping of the SAN that combined light microscopy and multiple microelectrode recordings to map AP timing across the tissue during each beat soon uncovered an apparent pathway of impulse propagation in multiple animal models, with APs originating in the central SAN between the superior and inferior venae cavae, expanding to the rest of the node concentrically and ultimately to the atria [57]. Preferential propagation towards the crista terminalis as opposed to the interatrial septum was hypothesized to be determined by different proportions of electrically coupled cells in the two regions [57]. Combined immunohistochemistry and electrophysiology experiments revealed that as cells grew distant from the central SAN they gradually displayed electrical and structural properties more similar to those of atrial cells, with gradually higher contractility and shorter APs [58].

Based on their computational modelling of hundreds of coupled cells, which they simulated by differential equations describing their transmembrane ion currents, Michaels *et al.* posited in 1987 that all pacemaker cells in the SAN intrinsically produce spontaneous electrical oscillations, and that their dynamic mutual entrainment and synchronization produce a consensus rhythm across the tissue [59]. The application of voltage-sensitive dyes to the study of SAN electrophysiology allowed the simultaneous ‘optical mapping' of all electric impulses in the tissue, and thus the discovery that signal transduction across the node is more complex than the concentric radial models had previously hypothesized and may depend on variable preferential pathways [60–62]. It became evident during optical mapping experiments that the site of earliest AP appearance in the SAN was not always in the classically designated central region and could even shift over the length of a recording over several hours—casting doubt on the concept of a rigid leading pacemaker site and leading to the concept of ‘multicentric' SAN activation [63]. In fact, shifts in the location of the earliest site of impulse appearance in the SAN have been observed in response to pharmacological perturbations, hinting at a mechanism for redundancy and robustness for SAN function [64]. This phenomenon had also been related to the ‘wandering pacemaker,' and is enabled by the SAN's relatively wide spread throughout the intercaval region of the right atrium [65].

The combination of optical mapping and histological analysis of the canine and human SAN led to the identification of distinct myofibre tracts that create sinoatrial conduction pathways through which SAN impulses are transmitted to the atria, as well as an electric ‘blocking zone' in the interatrial septum composed of coronary arteries, fat and connective tissue [66,67]. Case studies revealed that normal SAN activation of the atria depended on these sinoatrial conduction pathways to be intact, and that these may be sites of remodelling by cardiac disease states and ageing [68]. SAN conduction pathway abnormalities caused by aberrant intercellular signalling have been implicated in atrial fibrillation and heart failure [69].

### (b) Brain-like cytoarchitecture of the sinoatrial node

For decades immunolabelling and quantitative polymerase chain reaction (q-PCR) techniques had been employed on right atrial tissue samples to identify SAN-specific protein expression patterns relative to its surrounding myocardium. Most notably, a high expression of the HCN4 channel, responsible for the *I*_f_ pacemaker current and a lack of the connexin 43 channel associated with tight junctions and high electrical coupling among cells, had been noted as defining features of the central SAN [70]. It was not until recently, however, that the distribution of SAN cells with differing protein expression profiles has been comprehensively described across the whole SAN.

**Figure 2.**
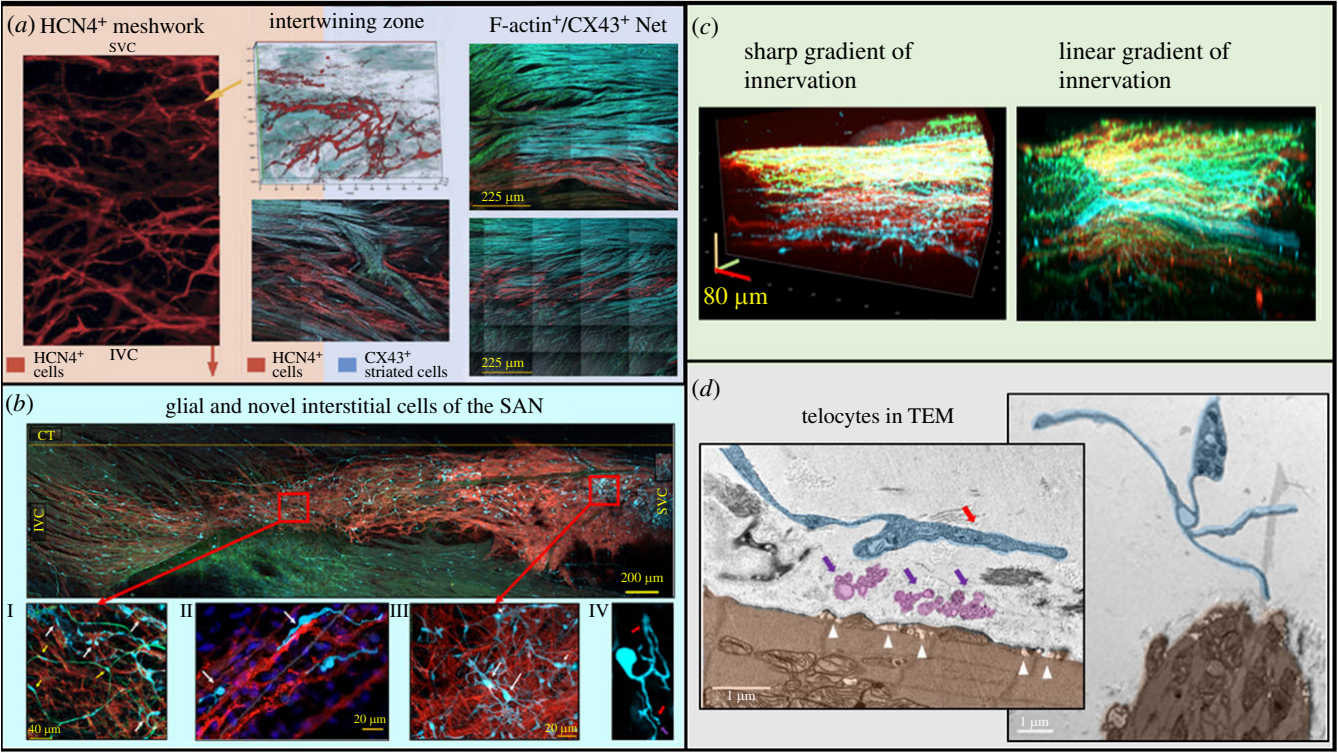
(*a*) The conduction pathway of SAN impulses discovered by confocal microscope dissection of immunolabelled mouse right atria: from the meshwork of HCN4^+^ cells (red) to the more organized network of F-actin^+^ (green)/CX43^+^ (blue) cells which transmit impulses to the atria. (*b*) A panoramic view of the mouse SAN obtained via tiled confocal imaging of the immunolabelled glial and interstitial cell populations of the node (S100B seen in blue, GFAP seen in green), alongside zoomed-in images of GFAP^+^ glial cells and S100B^+^/GFAP^−^ interstitial cells. SVC: superior vena cava; IVC: inferior vena cava; CT: crista terminalis. (*c*) Interstitial cells, identified by transmission electron microscopy (TEM), that appear to release vesicles (violet) towards pacemaker cells. (*d*) 3D reconstructions of different distributional patterns of neuronal projections, with tyrosine hydroxylase (TH) in green and vesicular acetylcholine transporter (VAChT) in blue. (*a*) Adapted from [20]; (*b*–*d*) adapted from [21].

Fortunately, the mouse SAN is sufficiently thin for it to be fully imaged from endocardium to epicardium, from superior vena cava to inferior vena cava and from the crista terminalis to the interatrial septum at high zoom, allowing visualization of the fine structure of the node via confocal tiling and 3D virtual slicing [21,71–73]. Detailed confocal imaging of intact mouse SAN preparations enabled the discovery of a population of densely interconnected HCN4-expressing pacemaker cells which extend across the node, described as a meshwork, owing to its structural heterogeneity (figure 2*a*,*b*) [20]. Pacemaker cells within the meshwork are interconnected by numerous, highly variable thin cellular extensions that had never been visualized previously. These branches approach the plasma membranes of nearby pacemaker cells, nerves, glial cells, blood vessels and more [21]. A lack of CX43 expression in HCN4^+^ cells suggests low electrical coupling [20]. The HCN4^+^ meshwork intertwines in specific tracts with a notably more regular network of highly coupled striated cells which coexpress CX43 and F-actin (figure 2*a*) [20]. This CX43^+^/F-actin^+^ (striated pacemaker cell) network does not intertwine homogeneously with the HCN4^+^ meshwork, but rather emits projections into it to form anatomical interactions with HCN4^+^ pacemaker cells (figure 2*a*). The defined sarcomeres of CX43^+^/F-actin^+^ pacemaker cells, as well as their formation within a highly structured network, suggest that, unlike cells in the HCN4^+^ meshwork, mechanical signal transduction may play a role in the pacemaking function of these highly electrically coupled cells. This CX43^+^/F-actin^+^ network of cells is hypothesized to mediate the transduction of the signal initiated within the HCN4^+^ meshwork of the SAN to the atria [20]. Thus, the spatial distribution and interactions among these cell types create three distinct zones of SAN cytoarchitecture: (i) the HCN4^+^ meshwork; (ii) the intertwining zone between the meshwork and CX43^+^/F-actin^+^ tracts; and (iii) organized CX43^+^/F-actin^+^ ‘highways' of cells that conduct signals from the meshwork to the atria.

The complexity of SAN structure and function does not end with different populations of pacemaker cells, however: many other cell types are present within the SAN and are active participants in impulse initiation and/or electric conduction to the right atrium. Single-cell RNA sequencing experiments and differentially expressed gene (DEG) analysis of isolated SAN cells have identified four major ‘clusters' of SAN cells based on functional gene coexpression profiles [74]. Higher-definition single nuclei RNA sequencing experiments, coupled with proteomic analysis, have identified 12 clusters of SAN cells, including neurons, endocardial and epicardial cells, epithelial cells, macrophages, myocytes, vascular endothelial cells, three types of adipocytes and two types of fibroblasts [37]. Further, about half of the SAN is composed of connective tissue, which has been implicated in the aetiology of heart failure and arrhythmias, because increased fibrosis impedes conduction from the node to the atria [75]. The fibroblasts within this connective tissue, although not electrically excitable, are also known to interact anatomically with pacemaker cells and possibly act as buffers for electrical conduction in the node [76]. Fibroblasts have also been found to alter metabolic rates in pacemaker cells, thus influencing their rhythmogenic activity [77].

Undoubtedly, of high importance among the cell types in the SAN are also the neuronal projections that emanate from the vagus nerve, paravertebral sympathetic network and parasympathetic trunk to create the intrinsic cardiac nervous system, which are responsible for the autonomic influence on heart rate and rhythm. Before reaching the SAN, autonomic nerves and their associated glial cells congregate in the cardiac ganglia located on the superior and dorsal surface of the right atrium, superior surface of the left atrium, and the tissue near the origin of the aorta and pulmonary trunk [78]. These ganglia receive and relay information to and from the heart, acting as important mediators between the brain and SAN [79]. Ultimately, projections emanating from the nerves of the cardiac ganglia penetrate the right atrium to form the neuronal plexus of the SAN. Many of these nerves form microcircuits in the intrinsic cardiac ganglia embedded among the fat pads on the outer surface of the right atrium [80]. Other than projections conveying neurotransmitter input to pacemaker cells and coronary vessels, the neuronal plexus also contains mechanosensitive and chemosensitive afferent nerves which inform the nervous system of cardiac blood pressure, volume and chemical composition [81]. Aberrations in the activity and architecture of the intrinsic cardiac nervous system are implicated in arrhythmias and are the target of many pharmacological and surgical treatments [82]. Of particular interest is the imbalance of signalling between the sympathetic and parasympathetic arms of autonomic input, a proposed model for the initiation of atrial fibrillation, which can be addressed by surgical, pharmacological or radiofrequency ablation of the vagus nerve or ganglionated plexus [83].

Recent experiments employing confocal microscopy and virtual slicing of immunolabelled mouse SAN preparations have added nuance to the study of the intrinsic cardiac nervous system (ICNS) by demonstrating in microscale 3D reconstructions the noteworthy heterogeneity of sympathetic and parasympathetic innervation within any given SAN as well as between those of different mice [21] (figure 2*c*). These variable distribution patterns create areas of dominant excitatory or inhibitory input, the functional implications of which are still to be uncovered [21].

It is important to note that the majority of the cells composing the cardiac neural plexus are not neurons, however, but rather peripheral glial cells, including Schwann cells and satellite glia, which are known to modulate the morphology and activity of SAN autonomic innervation [79,84]. The incompleteness of discussing brain structure and function without the inclusion of glial cells, often identified by their expression of markers including S100B and GFAP, has been realized for some time [85], and it is clear that full understanding of the cardiac nervous system will also require inclusion of these varied populations of cells. For instance, a recent study on zebrafish uncovered a population of astroglia-like peripheral glial cells immunolabelled by GFAP, termed ‘nexus glia', that impact on cardiac homeostasis by modulating the responses to both sympathetic and parasympathetic stimulation [86]. Genetically modified zebrafish unable to produce meteorin, a differentiation regulator required for nexus glia development, have a higher incidence of arrhythmia and ventricular fibrillation than wild-type [86]. The commonly used glial marker S100B, a secreted Ca^2+^ binding protein with both intra- and extracellular modes of action, has been subject of increased interest owing to its inhibitory effect on AP firing in isolated murine ICNS neurons, and its ability to stimulate neurite growth [87]. Further, higher plasma levels of S100B are associated with a reduction of recurrent atrial fibrillation in patients undergoing catheter ablation [87].

Bychkov *et al*. [21] in the attempt to describe glial cells other than the PGCs that follow autonomic nerves, have added additional complexity to the understanding of SAN cytoarchitecture via the discovery of a novel interstitial cell population in the murine SAN which expresses S100B, but not GFAP, that are present exclusively within the HCN4^+^/CX43^−^ meshwork (figure 2*b*). These S100B^+^/GFAP^−^ cells escape concise definitions by exhibiting varied morphology and distribution within and among SANs, as well as frequent yet heterogeneous connectivity with HCN4^+^ pacemaker cells, ICNS nerves and blood vessels (figure 2*b*). Cells with similar morphology to the S100B^+^/GFAP^−^ cells visualized by confocal microscopy have been identified by tranmission electron microscopy (TEM) as SAN ‘telocytes,' and have been noted to secrete vesicles towards pacemaker cells (figure 2*d*) [21,88].

### (c) Local calcium releases within and among sinoatrial node pacemaker cells

**Figure 3.**
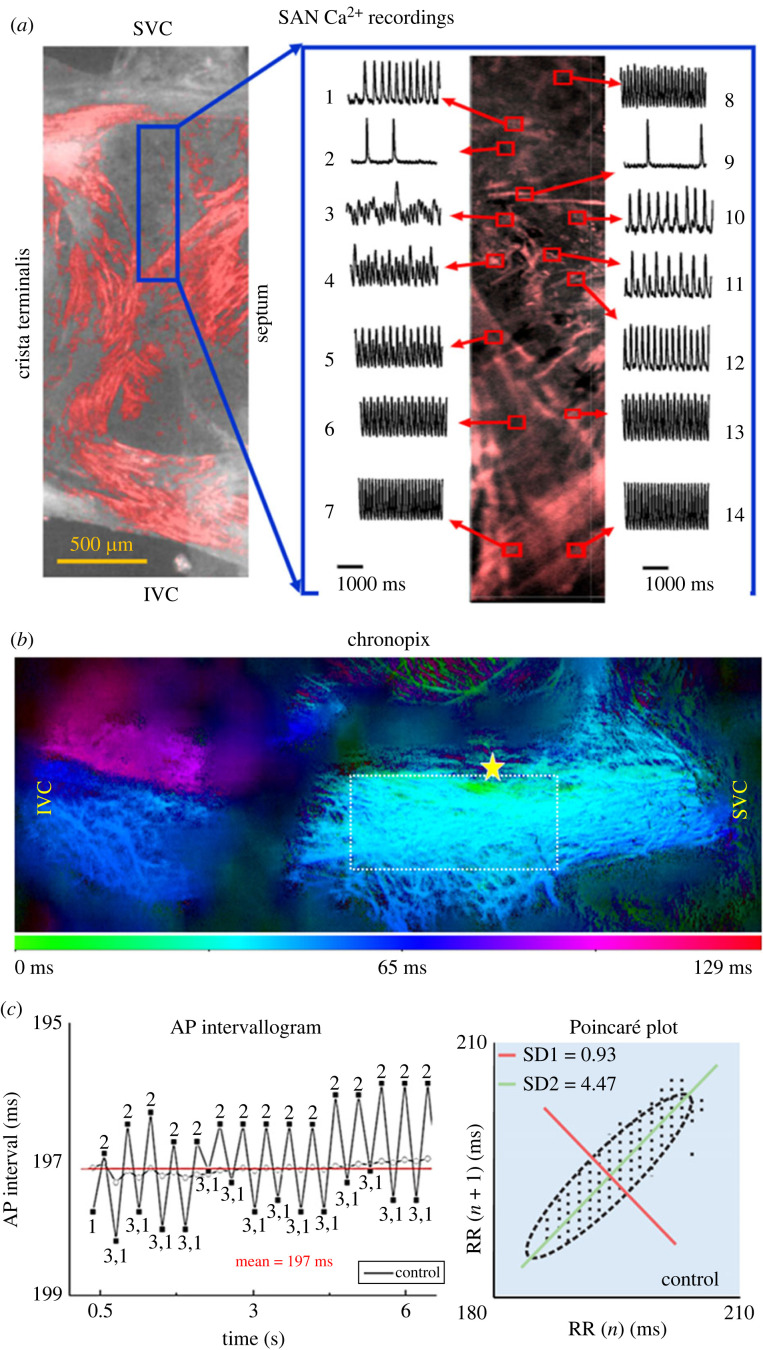
(*a*) A mouse SAN preparation imaged at 5× magnification, with red colour indicating Ca^2+^-induced Fluo-4 fluorescence during an action potential (AP) cycle. The blue box shows the quantification of heterogeneous Ca^2+^ intensity patterns in cells imaged with a 10× water-immersion objective from the indicated area of the SAN. (*b*) The order of AP-induced Ca^2+^ transient appearance throughout tissue during each SAN impulse is visualized via chronopix analysis. In (*c*), beat to beat variability in inter-AP intervals as measured by SAN atrial intracellular recordings is also shown (time series on the left). A Poincaré plot (right) of inter-AP intervals depicting short- (SD1) and long- (SD2) term correlations within the AP time series. (*a*) Adapted from [20]; (*b*,*c*) adapted from [21].

The heterogeneous cellular arrangement within the SAN sets the stage for the complex intracellular and intercellular signalling that leads to pacemaking. Ca^2+^ recordings using the Fluo-4 indicator in *ex vivo* SAN tissue preparations revealed that Ca^2+^ signalling within and among SAN cells is extremely variable: in any given SAN preparation some cells may exhibit rhythmic LCRs, similar to those observed in isolated SAN pacemaker cells [30], while other cells produce AP- induced calcium transients, and many exhibit both types of Ca^2+^ signals, with the timing relationship between the two also being variable (figure 3*a*; electronic supplementary material, video S2*b*). Comprehensive description of the Ca^2+^ signals in SAN tissue is facilitated by the construction of ‘chronopix', spatial and temporal maps of SAN cell activation in each pixel of panoramic (2.5× magnification) Ca^2+^ video recordings, enabling quantification of the phase shift or synchronicity among the times when intracellular and tissue-wide Ca^2+^ events become visible at low zoom (figure 3*b*) [20,21]. Recordings at higher magnification (10×) revealed that rhythmic LCRs in some cells precede AP-induced calcium transients, as in isolated SAN cells, while some clusters of cells exhibit exclusively LCRs that precede AP-induced calcium transient generation in nearby cells, which, themselves, lack LCRs (electronic supplementary material, video S2*b*) [20]. In many cases, cells exhibit LCRs with no discernible relationship to nearby AP-induced calcium transients [20]. AP-induced calcium transients in some cells within the SAN are not synchronized to the AP rate and rhythm recorded in the atria [20]. Thus, expansion of the frontier of knowledge about the heart's pacemaker requires new understanding of how these local, heterogeneous, Ca^2+^ behaviours within and among SAN cells become organized into a highly synchronized impulse that exits the SAN at fairly regular intervals to initiate the heartbeat [89,90].

Low-zoom (2.5× air objective) recordings of Ca^2+^ dynamics across the entire SAN HCN4^+^ meshwork can be produced using tissue preparations extracted from HCN4-GCaMP8 transgenic mice, which express a genetically encoded Ca^2+^ indicator exclusively within HCN4^+^ pacemaker cells (electronic supplementary material, video S2*c*). Chronopix analysis of such recordings has revealed that, over the course of a given impulse, the SAN can be separated into spatio-temporally distinguishable areas of Ca^2+^ signal appearance (figure 3*b*). The raw Ca^2+^ signals recorded in the SAN regions delineated by the chronopix exhibited different rates of rise, amplitudes and rates of decay. In addition, the tracings of these Ca^2+^ recordings displayed a regular pattern of long–short–long intervals between oscillations, which resonate above and below the *post hoc* calculated mean interval [21]. A recurring pattern of AP cycle-to-cycle variability (numbered 1–3 in figure 3*c*) can also be observed in intracellular AP recordings obtained from the right atrial tissue of SAN preparations. This phenomenon of inter-AP and inter-AP-induced calcium transient interval variability observed in SAN tissue recapitulates, at a higher level of complexity, the behaviour observed in isolated SAN pacemaker cells (figure 1; electronic supplementary material, videos S1*a*,*b*).

The phase shifts between the times of appearance of large-scale Ca^2+^ signals in these regions of the SAN are large enough such that the last area to activate over the course of a beat has not fully subsided by the time the earliest activity emerges for the beat after it. This may be another tissue-level mechanism that preserves the robustness of SAN activity: if impulses overlap and are not completely synchronized in time, there is no timepoint at which the entire SAN tissue is deactivated [21].

This heterogeneity, alongside the loose electrical coupling of cells in the meshwork of HCN4^+^/CX43^−^ cells in the central SAN, indicates that pacemaking is an emergent property of the entire tissue, irreducible to the activity of any given cell within it [20]. The self-ordering process by which spatio-temporally asynchronous signals in different areas of the tissue give rise to the relatively rhythmic impulse that exits the SAN is yet to be understood [89,90]. Part of the answer may be that neurotransmitter-mediated communication in the node is *not* limited to acetylcholine and adrenaline, as it also includes nitric oxide [79], glutamate [91], GABA [92], serotonin [93] and neuropeptide Y [79]. A more comprehensive characterization of the cell-to-cell communication in SAN architecture will require characterization of the cells producing these other neurotransmitters and their receptors. An additional factor may be heterogeneity in mechanical strain produced by the heterogeneous distribution of myocytes with organized sarcomeres in SAN tissue zones at and beyond the borders of the HCN4^+^ meshwork [20].

The discovery of S100B^+^/GFAP^−^ telocytes in the SAN (figure 2*b*), combined with S100B's Ca^2+^ buffering capacity, has stimulated investigations of the impact of S100B protein application on the rhythmicity of APs and Ca^2+^ activity in the SANs of HCN4-GCaMP8 mice [21]. Indeed, in recent experiments, S100B induced a reduction in the mean SAN AP-induced calcium transient firing rate, increasing inter-AP-induced calcium transient interval variability. Further, S100B altered the Ca^2+^ waveform properties within different SAN regions, significantly slowing their rates of rise and decay, but also eliminated AP-induced calcium transients from the area of earliest impulse emergence seen in control, such that the site of earliest AP-induced calcium transient appearance shifted inferiorly, similar to what is seen in response to acetylcholine [94]. In addition to peak-to-peak interval variability, beat-to-beat variability patterns in the time to peak, maximum rate of rise and maximum rate of decay of the whole-SAN ensemble Ca^2+^ signal were also distorted in the presence of S100B [21]. In intracellular AP recordings from the right atrium, S100B also slowed down the AP firing rate and distorted the reliable pattern of beat-to-beat variability [21].

The desynchronization of Ca^2+^ signalling, increased inter-AP interval variability and lower mean AP firing rate observed to result from S100B application is further evidence that these physiological events are tied by shared underlying regulatory mechanisms, as observed in isolated single pacemaker cells [27]. These effects of S100B on SAN function may provide a clue as to how the varied cell populations of the SAN may influence pacemaking in ways that greatly differ from the well-studied effects of autonomic input. Still, it is not yet understood how S100B or other signalling molecules interact with autonomic input to pacemaker cells.

The effects of S100B on SAN pacemaker cells, as well as the intimate anatomical interactions between S100B^+^/GFAP^−^ telocytes visualized in both immunolabelled preparations and TEM images, mirror the relationship of astrocytes to central nervous system (CNS) neurons. This similarity leads to the analogy of the SAN as the heart's ‘central brain' (electronic supplementary material, videos S2*a*,*b*) [21,95].

## 5. So, when and why will it beat?

Given that SAN cytoarchitecture and function mimic that of the brain (figures 2 and 3), we may now conceive of its function as defined by the interconnectivity of SAN ‘brain cells,' each with its intrinsic coupled-clock system that can be modulated by nervous input, mechanic stimulation, hormonal factors, etc., allowing them to fire APs at a variety of rates (*robustness*) and to change their firing rates rapidly (*flexibility*). Although most SAN cells studied in isolation can fire APs spontaneously, whether they will and at what rate depends upon the molecular expression and post-translational modifications of coupled-clock system proteins, both of which vary not only within and among individual pacemaker cells within a given SAN locus, but also among SAN loci. The main determinant of the rate and rhythm of the initiation of the ignition of electrochemical signals that effects spontaneous diastolic membrane depolarization is the SR Ca^2+^ load. The beat-to-beat levels of the Ca^2+^ load within the SR determines the periodicity of LCR emergence via RyRs. Short intervals are created when LCRs are robust and fully deplete the SR load (which consists of a form of Ca^2+^ load-dependent *memory* for the coupled-clock system), while long-interval LCRs are feeble and cause less depletion in single cells. The SR Ca^2+^ load can be impacted not only by neurotransmitter peptides, which impact the SR memory via effecting change on the phosphorylation state of clock proteins, but also by chemokines secreted from SAN glial cells and cytokines from immune cells.

The variable phosphorylation states of coupled-clock proteins among SAN cells leads to heterogeneity in phase, amplitude and frequency of electrochemical signals that they generate. To add to this complexity, the *in vivo* firing characteristics of SAN pacemaker cells also depend upon the specific temporal characteristics of intercellular communication, which are largely determined by the variable electro-mechanical coupling among SAN pacemaker cells, ranging from loose coupling (HCN4^+^/CX43^−^ cell meshwork) to very tight coupling (F-actin^+^/CX43^+^ network) (figure 2). Further, individual pacemaker cells are heterogeneous in the degree to which they receive chemical and anatomical input from other cell types of the SAN, including neurotransmitter-derived signals from nerves, chemokine and cytokine signals, peripheral glial cells, interstitial cells and immune cells (figure 3).

Because the heterogeneous signals originating within and among pacemaker cells that reside within the SAN are stochastic in nature, the SAN *cannot* act as a metronome. Rather, the SAN operates on a balance between the principles of criticality (Ca^2+^ clock) and limit-cycle oscillators (membrane clock) [27,96]. Thus, based on the degree of synchronicity achieved among *intra*cellular functions and *inter*cellular communication in the node within a given AP cycle, some AP intervals turn out to be shorter and others longer than the *mean rate* that the system is aiming for based on the body's need for blood flow in the physiological context (sleeping, lying down, standing, walking, mental or physical strain, etc.).

Paradoxically, although its activity resonates about a specific mean firing interval, the SAN is unaware of the specific mean it achieves within a time series, which is, in fact, a *post hoc* calculation made by an external observer. Thus, pacemaker cells, whether isolated or embedded in SAN tissue, are programmed to create a specific mean AP firing rate that does not exist on a beat-to-beat basis. Instead, the beat-to-beat decisions taken by pacemaker cells based on their intracellular phosphorylation states and intercellular communication can be best observed in the *running mean* of a beat time series. The running mean shows how the cell adjusts its near-term future intervals to hover about the mean prescribed by the physiological context.

The degree to which electrochemical signals within and among SAN pacemaker cells become synchronized over the course of multiple beats influences the variability in inter-AP intervals as well as mean AP frequency, as detected either by the inter-AP intervals measured by microelectrodes or by the EKG (PP or RR) intervals recorded on the body surface. Further, we have thoroughly described how sympathetic or parasympathetic input can, respectively, stimulate or inhibit coupled-clock system function and impact AP firing rate and rhythm. Thus, owing to its mechanistic relationship with autonomic surveillance, heart rate variability can be interpreted as the coordinated result of intrinsic SAN coupled-clock functions and their modulation by autonomic nervous system function.

## 6. The ‘SAN brain' is a *central player* within the hierarchy of the autonomic neuro-visceral surveillance network

The SAN, as the *heart's* ‘central brain,' is an active player within the autonomic neuro-visceral hierarchy that controls not only heart rate and rhythm, but also the automatic breathing rate and rhythm, as well as variations in blood pressure. Although the SAN is informed and modulated by autonomic signals, its multiple anatomically entrained cell populations provide it the necessary complexity for it to function *independently*, i.e. in the *absence* of neuronal input from the CNS brain. However, there is one principal distinction between the CNS and SAN brains: only the SAN contains myocytes with fully developed sarcomeres and tight electrical junctions, which may act as efferent fibres from the node. This new conceptualization of SAN structure and function also provides an answer to the myogenic versus neurogenic debate which has defined the study of cardiac rhythmogenesis for 150 years (for review see [58]): although SAN pacemaker cells have the ability to spontaneously generate rhythmic APs, SAN function at the tissue level *requires* brain-like cytoarchitecture.

It is well known that the SAN receives modulatory input from the dense network of autonomic neurites emanating from extracardiac and intracardiac ganglia (ICG), referred to as the 'little brain on the heart' [97], which fine-tunes the SAN's rhythmogenic function to meet the needs of the body for blood flow and pressure. The body informs the autonomic centres within the CNS brain of its needs for blood flow and pressure via sensory input from visual, olfactory and auditory signals that converge within the superchiasmatic nucleus, as well as mechanical and chemical signals arising within the heart and blood vessels. These signals are relayed from the brain stem/spinal cord, which initiate reflex neuronal inputs that are conducted via the ICG to the SAN autonomic plexus, which surrounds and embraces the ‘neuronal-like' HCN4^+^ cells within the heart's SAN brain. This neurotransmitter input modulates the same molecular functions that regulate the intrinsic pacemaker clocks within single SAN cells in the absence of autonomic input.

The intrinsic cellular ‘machinery' of pacemaker cells (i.e. the coupled-clock system) is equipped to rapidly decode autonomic input signals and compute the required transfer functions within its clock molecules to alter the clock system output, ensuring that timing of the subsequent impulses it generates match autonomic instructions. Autonomic input can be received and implemented on a beat-to-beat basis, but can also be applied over the course of several beats [27]. The adjusted impulses are then transmitted in parallel at millisecond to second time scales from the SAN to at least three targets: (i) the atria; (ii) the AV node, directly connected to the inferior part or ‘tail' of the SAN, from which, after a delay, impulses are conducted via the SAN brain axon-like cardiac conduction system (His bundle and bundle branches, and Purkinje fibres) to excite ventricular myocytes; and (iii) *efferent* communication back to the ICG neurons (a.k.a. *afferent* signals to the ICG), which relay the SAN central brain's message to the spinal cord, to the brain stem and to higher centres within the CNS, informing neurons within these structures of the fidelity of SAN reactions to neuronal input commands. Such communication from the heart to the ICG not only might occur via the autonomic neurites of the SAN's neural plexus [97], but may also be mechanical in nature, as each heartbeat generates stretching and vibrations on the epicardial surface that the mechanosensitive neurons of the ICG can then transfer to the spinal cord, brainstem and cortex [98,99]. Based on these signals arising from the SAN, CNS structures then create transfer functions aiming to synchronize them with other signals communicated to it from other components within the autonomic neuro-visceral axis. Ultimately, new instructions from the brain are broadcast throughout the autonomic neuro-visceral surveillance network to maintain coordinated resonance of blood pressure, heartbeats and breathing rates and rhythms. As a result, a beautifully tuned resonant coregulation of heart rate and rhythm, respiration rate and blood pressure at different time intervals emerges from the heart–brain autonomic surveillance network, informing on the health status of the body [47]. Disease states, and advancing age, even in the absence of disease [47], induce alterations in either the ‘heart's SAN brain' molecular machinery (coupled-clock system) or the efficacy of autonomic modulation of this machinery that become manifest as altered heart-rate variability [47], which creates discordance within the harmony that resonates throughout the autonomic neuro-visceral surveillance network.

## Data Availability

The data are provided in the electronic supplementary material [100].
